# Pulmonary hemodynamic disorder in pediatric sepsis and their correction with L-arginine infusion

**DOI:** 10.1186/cc12119

**Published:** 2013-03-19

**Authors:** M Georgiyants, V Korsunov

**Affiliations:** 1 Kharkov Medical Academy Post-Graduate Education, Kharkov, Ukraine

## Introduction

The acute respiratory distress syndrome and pulmonary hypertension (PH) is one of the factors of septic mortality. Some data demonstrate arginine deficiency as an important pathogenic factor of septic PH. We suppose that intravenous L-arginine infusion improves NO production and reduce PH.

## Methods

We examined 46 patients with sepsis, severe sepsis and septic shock in accordance with San Antonio Criteria. Organ dysfunction severity was defined according to SOFA. The mean pulmonary artery pressure (MPAP) was estimated by Doppler method, cardiac output (CO), and stroke volume (SV) - by ultrasound M-mode. SpO_2_, mean arterial pressure (MAP), blood gas, and plasma NO level was evaluated. The patients of Group 1 (*n = *21, age 22.1 ± 8.5 months) had respiratory and hemodynamic support, and antibiotics. Group 2 (*n = *25, age 27.0 ± 11.2 months) had the same treatment with continuous i.v. infusion of 5 ml/kg body weight 4.2% L-arginine solution during 24 hours.

## Results

The patients of Groups 1 and 2 did not have statistical difference of SOFA (4.1 ± 1.0 vs. 4.4 ± 0.7), need for ventilator support (57.0 ± 11.0% vs. 44.0 ± 10.0%), and dose of inotropes (*P >*0.05). The reduction of MPAP and increase of SaO_2 _was significantly higher in Group 2 compared with Group 1 (*P <*0.001). The increase in plasma NO level was significantly higher in Group 2 (Table 1).

**Table 1:** Hemodynamic parameters before and after Treatment

Value	Group 1 before	Group 1 after	Group 2 before	Group 2 after
MAP (mmHg)	66.9 ± 3.4	68.8 ± 3.0	72.8 ± 2.0	71.8 ± 1.3
MPAP (mmHg)	43.5 ± 2.6	44.3 ± 3.4	50.8 ± 3.0	28.8 ± 2.1
CO (l/minute/m^2^)	4.2 ± 0.2	5.0 ± 0.4	4.8 ± 0.2	4.8 ± 0.3
SpO_2 _(%)	93.7 ± 1.2	93.4 ± 1.5	94.6 ± 0.7	98.1 ± 0.3

## Conclusion

L-Arginine i.v. infusion 5 ml/kg during 24 hours increased NO blood level and decreased MPAP in pediatric sepsis, but did not deteriorate hemodynamic system values.

